# Supplementary material for: The Systems Biology Research Tool: evolvable open-source software
Source: BMC Syst Biol. 2008 Jun 29;2:55. doi: 10.1186/1752-0509-2-55 (PMC2446383; doi:10.1186/1752-0509-2-55)
Supplement: Additional file 1 — SBRT Archive. An archive of the current version of the Systems Biology Research Tool. [file 1752-0509-2-55-S1.zip › sbrt-1.4.0/doc/developers_guide/api/sbrt/kernel/math/algebra/MatrixElement.html]

MatrixElement


|  |  |  |
| --- | --- | --- |
| **Overview** \| **Package** \| **Class** \| **Use** \| **Tree** \| **Deprecated** \| **Index** \| **Help** | |  |
| **PREV CLASS**   **NEXT CLASS** | **FRAMES**    **NO FRAMES**     **All Classes** |  |
| SUMMARY: NESTED \| FIELD \| CONSTR \| METHOD | DETAIL: FIELD \| CONSTR \| METHOD |  |


---


## sbrt.kernel.math.algebra Class MatrixElement<R,C,E>

```
java.lang.Object
  sbrt.kernel.math.algebra.MatrixElement<R,C,E>
```

**Type Parameters:**: `R` - the row index type.: `C` - the column index type.: `E` - the matrix element type.

---

``` public final class MatrixElement<R,C,E> extends java.lang.Object ```

This class is used to represent matrix elements.

**Author:**
:   This class was written and documented by
    Jeremiah Wright while in the Wagner lab.

---

**Constructor Summary**

`MatrixElement(IndexPair<R,C> indices,
U element)`
  
          Constructs a new matrix element.

`MatrixElement(S rowIndex,
T columnIndex,
U element)`
  
          Constructs a new matrix element.


| **Method Summary** | |
| --- | --- |
| `boolean` | `equals(java.lang.Object obj)` |
| `C` | `getColumnIndex()`             Returns the column index of this matrix element. |
| `E` | `getElement()`             Returns the value of this matrix element. |
| `IndexPair<R,C>` | `getIndices()`             Returns the pair of indices. |
| `R` | `getRowIndex()`             Returns the row index of this matrix element. |

**Methods inherited from class java.lang.Object**

`clone, finalize, getClass, hashCode, notify, notifyAll, toString, wait, wait, wait`

**Constructor Detail**

### MatrixElement

```
public MatrixElement(S rowIndex,
                     T columnIndex,
                     U element)
```

:   Constructs a new matrix element.

    **Type Parameters:**: `S` - a type extending the primary row type.: `T` - a type extending the primary column type.: `U` - a type extending the primary element type. **Parameters:**: `rowIndex` - the row index.: `columnIndex` - the column index.: `element` - the matrix element at the specified row and column indices.

---


### MatrixElement

```
public MatrixElement(IndexPair<R,C> indices,
                     U element)
```

:   Constructs a new matrix element.

    **Type Parameters:**: `U` - a type extending the primary element type. **Parameters:**: `indices` - the row and column index of this element.: `element` - the matrix element at the specified row and column indices.


**Method Detail**

### getRowIndex

```
public R getRowIndex()
```

:   Returns the row index of this matrix element.

    :   **Returns:**: the row index of this matrix element.

---


### getColumnIndex

```
public C getColumnIndex()
```

:   Returns the column index of this matrix element.

    :   **Returns:**: the column index of this matrix element.

---


### getIndices

```
public IndexPair<R,C> getIndices()
```

:   Returns the pair of indices.

    :   **Returns:**: the pair of indices.

---


### getElement

```
public E getElement()
```

:   Returns the value of this matrix element.

    :   **Returns:**: the value of this matrix element.

---


### equals

```
public boolean equals(java.lang.Object obj)
```

:   **Overrides:**: `equals` in class `java.lang.Object`


---


|  |  |  |
| --- | --- | --- |
| **Overview** \| **Package** \| **Class** \| **Use** \| **Tree** \| **Deprecated** \| **Index** \| **Help** | |  |
| **PREV CLASS**   **NEXT CLASS** | **FRAMES**    **NO FRAMES**     **All Classes** |  |
| SUMMARY: NESTED \| FIELD \| CONSTR \| METHOD | DETAIL: FIELD \| CONSTR \| METHOD |  |


---
